# Metagenomic Characterization of Microbiome Taxa Associated with Coral Reef Communities in North Area of Tabuk Region, Saudia Arabia

**DOI:** 10.3390/life15030423

**Published:** 2025-03-07

**Authors:** Madeha O. I. Ghobashy, Amenah S. Al-otaibi, Basmah M. Alharbi, Dikhnah Alshehri, Hanaa Ghabban, Doha A. Albalawi, Asma Massad Alenzi, Marfat Alatawy, Faud A. Alatawi, Abdelazeem M. Algammal, Rashid Mir, Yussri M. Mahrous

**Affiliations:** 1 Department of Biology, Faculty of Science, University of Tabuk, Tabuk 71491, Saudi Arabia; a_alotaibi@ut.edu.sa (A.S.A.-o.); b.alharbi@ut.edu.sa (B.M.A.); dalshehri@ut.edu.sa (D.A.); h_ghabban@ut.edu.sa (H.G.); d.albalawi@ut.edu.sa (D.A.A.); amalenzi@ut.edu.sa (A.M.A.); mevalatawi@ut.edu.sa (M.A.); falatawi@ut.edu.sa (F.A.A.); 2 Biodiversity Genomics Unit, Faculty of Science, University of Tabuk, Tabuk 71491, Saudi Arabia; 3 Department of Bacteriology, Immunology, and Mycology, Faculty of Veterinary Medicine, Suez Canal University, Ismailia 41522, Egypt; abdelazeem.algammal@vet.suez.edu.eg; 4 Prince Fahd Bin Sultan Research Chair for Biomedical Research, Department of Medical Lab Technology, Faculty of Applied Medical Sciences, University of Tabuk, Tabuk 71491, Saudi Arabia; rashid@ut.edu.sa; 5 Department of Science and Basic Studies, Applied College, University of Tabuk, Tabuk 71491, Saudi Arabia; y.mahrous@ut.edu.sa

**Keywords:** coral reef, microbiome, sea sediment, bacterial diversity, microbial ecology, coral-associated bacteria, Red Sea, 16S rRNA

## Abstract

The coral microbiome is highly related to the overall health and the survival and proliferation of coral reefs. The Red Sea’s unique physiochemical characteristics, such a significant north–south temperature and salinity gradient, make it a very intriguing research system. However, the Red Sea is rather isolated, with a very diversified ecosystem rich in coral communities, and the makeup of the coral-associated microbiome remains little understood. Therefore, comprehending the makeup and dispersion of the endogenous microbiome associated with coral is crucial for understanding how the coral microbiome coexists and interacts, as well as its contribution to temperature tolerance and resistance against possible pathogens. Here, we investigate metagenomic sequencing targeting 16S rRNA using DNAs from the sediment samples to identify the coral microbiome and to understand the dynamics of microbial taxa and genes in the surface mucous layer (SML) microbiome of the coral communities in three distinct areas close to and far from coral communities in the Red Sea. These findings highlight the genomic array of the microbiome in three areas around and beneath the coral communities and revealed distinct bacterial communities in each group, where *Pseudoalteromonas agarivorans* (30%), *Vibrio owensii* (11%), and *Pseudoalteromonas* sp. Xi13 (10%) were the most predominant species in samples closer to coral (a coral-associated microbiome), with the domination of *Pseudoalteromonas_agarivorans* and *Vibrio_owensii* in Alshreah samples distant from coral, while *Pseudoalteromonas*_sp._Xi13 was more abundant in closer samples. Moreover, Proteobacteria such as *Pseudoalteromonas*, *Pseudomonas* and *Cyanobacteria* were the most prevalent phyla of the coral microbiome. Further, Saweehal showed the highest diversity far from corals (52.8%) and in Alshreah (7.35%) compared to Marwan (1.75%). The microbial community was less diversified in the samples from Alshreah Far (5.99%) and Marwan Far (1.75%), which had comparatively lower values for all indices. Also, *Vibrio* species were the most prevalent microorganisms in the coral mucus, and the prevalence of these bacteria is significantly higher than those found in the surrounding saltwater. These findings reveal that there is a notable difference in microbial diversity across the various settings and locales, revealing that geographic variables and coral closeness affect the diversity of microbial communities. There were significant differences in microbial community composition regarding the proximity to coral. In addition, there were strong positive correlations between genera *Pseudoalteromonas* and *Vibrio* in close-to-coral environments, suggesting that these bacteria may play a synergistic role in Immunizing coral, raising its tolerance towards environmental stress and overall coral health.

## 1. Introduction

Coral reefs are the globe’s most diversified symbiotic ecosystem. Corals coexist in a complex, multipartite symbiosis, with a variety of bacteria from different kingdoms, some of which are linked to essential processes like climate change adaptation [1]. Further, significant corals function as meta organisms reliant on dynamic multipartite symbioses with various microbes. Homeostasis is maintained within this intricate system by these interkingdom interactions between the multicellular eukaryotic coral host and its accompanying microbiota, which have supported its resilience for more than 500 million years [2,3,4]. Associations within the meta organism comprise a large diversity of viruses, prokaryotes, and microeukaryotes that are collectively termed the coral holobiont [5,6,7,8]. Chief among the holobiont microbes, the primary endosymbiotic dinoflagellate of the family *Symbiodiniaceae* provides the bulk of the required nutritional needs to their coral hosts [9,10]. In addition, an increasing body of evidence is unravelling the key roles particular bacterial species in specific and general prokaryotic communities play in maintaining holobiont fitness, potentially via exchanging essential metabolites, recycling nutrients, and providing protection against pathogenic microbes [11]. In the Anthropocene era, climate change disrupts these symbiotic relationships, leading to dysbiosis that is characterized by the overgrowth of opportunistic and putatively pathogenic microbes and results in a compromised coral immune system, inevitably causing the onset of coral bleaching and/or disease [12]. Most coral microbiome work has been exclusively focused on either endosymbiotic algae or bacteria while ignoring the other, largely underexplored members of the coral microbiomes due to difficulties associated with studying their role in the holobiont. However, our comprehension of the nature and functional relevance of intricate symbiotic connections within corals is limited by information gaps and technological difficulties. Despite the fact that corals collectively contain one-third of all marine bacterial phyla, known bacterial symbionts and antagonists of corals only make up a small portion of this diversity. These taxa cluster into specific genera, indicating that these bacteria were able to acquire a niche within the holobiont through selective evolutionary mechanisms.

### 1.1. Diverse Bacterial Symbionts Associated with Corals

Corals harbor a diverse bacterial microbiome [13], spanning 39 phyla [14], over one-third of the bacterial phyla identified in seawater [15]. A component of these coral-associated bacterial communities is believed to enhance the health and resilience of corals [16,17,18]. In the extensive array of bacterial phyla linked to corals, *Proteobacteria*, *Bacteroidetes*, *Cyanobacteria*, and *Firmicutes* rank as some of the most prevalent, as determined by the 16S rRNA gene phylogeny analysis of 21,100 sequences obtained from a public database [14]. Furthermore, the majority of cultivable bacteria were found to belong to the phyla Proteobacteria, *Firmicutes*, *Bacteroidetes*, and *Actinobacteria* in a recent meta-analysis of 3055 bacterial isolates from 52 coral investigations and provides valuable microbial resources to maintain the stability of coral ecosystems and investigate their roles in the marine carbon cycle [19]. Environmental stresses like ocean warming may cause the collective reef microbiome to react quickly, which might ultimately result in reef microbialization. Reef microbialization is marked by a change in both abundance and biomass favoring microbes, particularly a transition towards a pathogenic assemblage that has the potential to cause significant declines [20]. Coral-associated bacteria inhabit several compartments within the coral, such as the SML, tissues, gastric cavity, and skeleton [12,21,22]. Unique physiochemical characteristics and environmental gradients, such as pollution [23,24], significantly influence the microbial composition inside these compartments [21,25,26]. The bacterial makeup differs among these several niche compartments, with certain bacteria favoring the colonization of certain compartments. Bacteria from the families *Chloroflexi*, *Sphingobacterium*, *Roseobacter*, and *Pseudoalteromonas* were exclusively identified in the SML [25], whilst *Endozoicomonas* was located among aggregates in coral tissues [27,28]. This niche specificity implies that some bacteria are tailored to the local microenvironment inside the coral colony, which eventually results in distinct interactions with the host within each milieu. There are a variety of host-specific bacteria that are connected with corals, but there are also a small number of bacterial phylotypes that show signs of a long-term host-microbe association, particularly with Endozoicomonas. If host-microbe cophylogeny is likely to play a role in phylosymbiosis, other factors, such as biogeographic influences, also play an important role in shaping this pattern. This is supported by recent findings, which further support the hypothesis that biogeographic influences are just one of many variables that shape the pattern of phylosymbiosis. The varying levels of cophylogeny between coral microbes and their hosts also highlight the fact that the microbiome is not a single entity that is subject to uniform selection, and that the holobionts’ abundance is not correlated with their shared evolutionary history. Instead, it comprises a multitude of distinct participants with varying degrees of historical association with both the host and each other. [21].

### 1.2. Function of Coral-Associated Microbiome in Coral Life and Health

The coral–Symbiodiniaceae symbiosis is the driving force of the holobiont, and this symbiosis interaction can shape the whole coral life and development. The symbiotic association between corals and Symbiodiniaceae facilitated the formation of the reef (calcium carbonate skeleton) through reciprocal nutrients [29]. This symbiosis depends on mutual metabolite exchanges, wherein Symbiodinaceae provide surplus photosynthetically produced dissolved organic matter to the coral host in return for access to inorganic nutrients and CO_2_ produced during respiration [9,30,31]. The transfer of organic photosynthates, such as glucose, by Symbiodiniaceae is energetically strong enough for the host to fulfill 100% of its respiratory needs [11,32]. While corals may assimilate ammonium for nitrogen acquisition, the Symbiodiniaceae primarily facilitate the absorption of inorganic nitrogen in the forms of nitrate and ammonium [33]. A fraction of this nitrogen is exchanged with the coral host as dissolved organic nitrogen (e.g., amino acids) [34,35,36].

### 1.3. Role of Nitrogen-Fixing Microbiome Associated with Corals

Nitrogen-fixing microbiomes are very common in coral [1]. Diazotrophs are frequently linked to coral tissues [5,33,37], especially during early life stages (larvae and juveniles) [38], suggesting the potential significance of nitrogen fixation within the coral holobiont. Communities of ammonia-oxidizing bacteria and archaea may partially oxidize ammonium produced by nitrogen fixation. Similarly, denitrifying bacteria have been documented in corals [39]. Rädecker et al. (2022) more recently documented the close connection between coral bleaching and disruptions in the nitrogen cycle [40]. Nonetheless, the molecular processes linking the nitrogen-related activities of these microbial communities remain mainly unidentified. Like all phytoplankton, *Symbiodiniaceae* form associations with bacteria that affect their physiology and the availability of nutrients [41]. Numerous *Symbiodiniaceae* cultures and other phytoplankton lineages share members of the *Rhodobacteraceae* family, which has been demonstrated to be crucial in supplying phytoplankton with vital nutrients, hormones, and cofactors [42].

### 1.4. Coral-Associated Microbiome in Red Sea

Microorganisms are essential in several reef processes, encompassing primary production and the cycling of nutrients and organic waste [43]. Microbes are pervasive symbionts of eukaryotic species, supplying the host with nutrition, facilitating chemical cycle, and offering defense roles [44]. Because of its unique physiochemical characteristics, including a significant north–south temperature and salinity gradient, the Red Sea is an especially intriguing research system. While the southern and western parts of the Red Sea are still completely unknown, the majority of research has been conducted in the center and northern sections [16,44,45]. In spite of the Red Sea’s enormous coral variety, *Pocillopora verrucosa*, *Dipsastraea* spp., *Pleuractis granulosa*, and *Stylophora pistillata* are the most researched corals [44]. The most common bacterial families include *Rhodobacteraceae* and *Vibrionaceae*, whereas bacteria from the class Gammaproteobacteria dominate microbial diversity [44,46]. The dominant north–south environmental conditions do seem to be correlated with the microbial population in the water column. For instance, the *cyanobacteria Synechococcus* and heterotrophic picoplankton are often more prevalent in the warmer, less salinized waters of the south. Conversely, the microorganisms linked to corals appear to be preserved across the Red Sea and several other regions globally [45,46]. Numerous coral species in the Red Sea have *Endozoicomonas bacteria*, a phenomenon also documented globally. Red Sea corals exhibit many microbial-based illnesses, such as white syndromes, skeletal eroding band, black band disease, and growth abnormalities; nevertheless, these occurrences are seldom in Red Sea waters [47,48,49]. Consequently, although significant climatic extremes influence free-living microbial communities in the Red Sea [48], the microorganisms in strictly regulated symbiotic habitats seem to be preserved, but strain-level genotype specialization remains a subject of ongoing investigation. In this research, we suppose that the microbiome’s role is to preserve the Red Sea ecosystem and its interactions with various environmental stressors and changes [49,50]. We predict that an integrated genetic survey of coral reef microbiomes, saltwater pools, and sediments will provide a better understanding of the symbiotic mechanisms and relationships between corals and the microbiome by tracking microbial diversity and function over time, with an emphasis on marine and coastal areas, emphasizing the significance of microbial genes, including their metabolic activities, adaptive strategies, symbiotic interactions, and roles in the carbon and nitrogen cycle. How is the extensive metagenomic research now being undertaken in the Red Sea enhancing our understanding of the coral-associated microbiome, and how can we rehabilitate the degraded coral communities by modifying their microbiome communities? In this study, we aim to investigate the coral-associated bacterial taxa and gene-forming clusters of microbiomes and their distribution along the environmental gradient in the studied sites. Further, we will identify the most prevalent bacterial taxa in proximity to coral communities and at further distances to enhance our understanding of the molecular processes behind coral–microbiota interactions related to temperature tolerance and potential pathogens causing diseases.

## 2. Materials and Methods

### 2.1. Study Location and Sampling

Samples of soil were taken from ten different places along the shore of three distinct sites in the Red Sea: Alshreah, Saweehal, and Marwan. Figure S1 displays the research area’s location on a map (Alshreah, Saweehal, and Marwan). Three duplicate soil samples were gathered from two habitats at each location, obtained far from coral and samples taken near to coral from different ten distinct sites in the selected areas. The coordinates of the selected site were 28°01′52.3″ N 34°39′22.1″ E for Saweehal, 29°01′33.7″ N 34°50′50.4″ E for Marwan, 29°06′41.7″ N 34°52′32.0″ E for Alshreah 1, and 29°05′22.7″ N 34°52′24.4″ E for Alshreah 2. The selected samples were collected at different depths from 10 m to 20 m. The samples were collected in sterile plastic containers and transported on ice to the University of Tabuk, Saudi Arabia’s Faculty of Science, Biodiversity Genomics Unit. After being stored at −80 °C for DNA extraction and quantification, the samples were sent to the Genome Life Science Company in India for metagenomics and DNA sequencing.

### 2.2. Isolation and Quantitative Analysis of DNA

DNA was extracted from the soil samples in accordance with the manufacturer’s instructions, utilizing an DNeasy PowerSoil Pro Kit (50) (Cat. No 47014) Qiagen (Germantown, MD, USA). The extracted DNA was then quantified using a NanoDROP 1000 from Thermoscientific (Waltham, MA, USA; Boston, MA, USA).

16S rRNA amplification: The DNA serves as a template for PCR, which amplifies a 500–1500 bp section of the 16S rRNA gene sequence. The 16S rRNA gene was amplified using universal primers 8F and 1492R. Any bacterium can have the region amplified by using universal primers that complement conserved areas. The QiaQick PCR purification kit from the Qiagen (Germantown, MD, USA) and Microcon-100 Microconcentrator columns (Millipore) are two excellent commercial kits that are available for purifying PCR products in order to eliminate superfluous primers and nucleotides. All PCR assays were carried out in reaction mixtures containing 16S rRNA universal primers 8F and 1492R (0.5 μM each), dNTP (0.2 mM), Taq polymerase (Fermentas), (Waltham, MA, Boston, MA, USA) (1.25 U), with the supplied buffer, MgCl2 (1.5 mM), template DNA (10 μL), adjusted with water to a final volume of 50 μL. The cycling parameters were: 94 °C for 7 min; 35 cycles of 94 °C for 30 s, 55 °C for 30 s, 72 °C for 60 s, and 72 °C for 10 min. The amplicons (1.5 kbp) were produced and seen on 1.8% (*w*/*v*) agarose gel electrophoresis.

### 2.3. Setting Up the Library

Following the manufacturer’s guidelines, libraries for paired-end sequencing were constructed utilizing a DNA Library Kit from Twist Bioscience for Illumina® (CAT 104119). South San Francisco HQ. 681 Gateway Blvd South San Francisco, CA 94080.

Initially, 50 ng of DNA was enzymatically sheared into smaller fragments, which were then prepared for adapter ligation through A-tailing and end repair. The ends of the DNA fragments were fitted with an Illumina-specific adapter to facilitate the binding of the sequencing primers, PCR amplification, and library formation. High-fidelity PCR amplification was performed using a HiFi-PCR MasterMix (Takara, Kusatsu, Japan) to maximize yield. The quantity and quality of the amplified libraries were evaluated using an Agilent Tape Station (4150) system (Agilent Technologies, Waldbronn, Germany) with High-Sensitivity (D1000) Screen-Tape^®^, following the manufacturer’s instructions.

### 2.4. Cluster Generation and Sequencing

After analyzing the Tape Station profile and determining the Qubit concentration, the library was placed into an Illumina NovaSeq 6000 system to generate clusters and sequence them. Paired-end sequencing was employed to sequence template fragments in both directions. Molecules from the library hybridized to corresponding adaptor oligonucleotides on the paired-end flow cell. Sequencing from the opposite end of the fragment was made possible by the adaptor design, which enabled selective cleavage of forward strands following reverse-strand re-synthesis.

### 2.5. Data Generation

To isolate individual samples, raw sequence data produced by the NovaSeq6000 platform (San Diego, CA, USA) were demultiplexed. Prior to de novo assembly, adaptor sequences and low-quality reads (QV < 20) were eliminated from the dataset through quality filtering. MEGAHIT v1.2.9 [51,52], a specialist metagenome assembler made to handle big and complicated metagenomic datasets, was then used to put the cleaned reads together.

### 2.6. Gene Prediction

Using Prodigal (v2.6.3) [53,54] in the metagenome gene prediction mode, gene prediction was executed on the assembled scaffolds. Following that, the projected gene sequences were used for functional and taxonomic studies.

#### 2.6.1. Metagenomic Sequencing and Analysis

Low-quality and single-ended metagenomic reads (length < 50 bp or with a quality value (Q-score) < 20) were removed using Sickle (v1.33) to ensure high-quality input data [55]. Subsequently, Multiple_Megahit program was used to assemble contigs and scaffolds for each sample, using default parameters and setting a minimum contig size of 300 bp. MetaGene-predicted open reading frames (ORFs) with a length ≥ 100 bp were translated into amino acid sequences. To construct a non-redundant gene catalog, gene sequences were clustered with identity ≥ 0.9 and coverage ≥ 0.9 using CD-HIT to eliminate redundancy. Using SOAP aligner, gene abundance was computed against the non-redundant gene catalog after it was mapped from high-quality reads. Taxonomic and functional annotations used BLASTP (BLAST v2.2.28+) to search the NR database with an e-value ≤ 1 × 10^−5^ and the KEGG database.

#### 2.6.2. Taxonomic Annotation

The taxonomy of metagenomic reads was assigned using Kaiju, a fast and sensitive classifier designed for metagenomes [56]. Kaiju relies on the Burrows–Wheeler transform algorithm to identify MEMs at the protein level within a reference database comprising annotated protein-coding genes. The database comprised complete microbial genomes from NCBI RefSeq, as well as the microbial subset of the NCBI NR database, optionally including fungi and microbial eukaryotes.

The reads were translated into all six possible reading frames and searched against the reference database for MEMs. Taxonomic assignments were completed using the lowest common ancestor (LCA) approach within the taxonomic hierarchy for reads that matched multiple sequences. This experiment used the standalone version of Kaiju with the following parameters: database: NR; sequence low-complexity filter: ON; run mode: Greedy; minimum match length: 11 amino acids; minimum match score: 75; and allowed mismatches: 5. The generated sequences were subsequently analyzed using Kaiju to provide high-quality taxonomic annotation.

#### 2.6.3. Alpha Diversity Analysis

Alpha diversity metrics, which quantify species richness and evenness within a sample, were calculated from OTU abundance data based on Kaiju classifications. For alpha diversity estimation, the phyloseq R package (v1.48.0) was used, applying the estimate richness function [57]. This analysis included indices, such as Chao1, ACE, Shannon, Simpson, Inverse Simpson, and Fisher, providing a detailed understanding of microbial diversity.

#### 2.6.4. Statistical Analysis and Data Visualization

All statistical analyses were carried out in R (v4.3.2), using various packages for data manipulation and visualization. Used packages and their versions were as follows: tidyverse (v2.0.0) for data manipulation [58], ggplot2 (v3.4.4) for making publication-quality plots [59], corrplot (v0.92) for visualization of correlation matrices, Complex Heatmap (v2.18.0) for making intricate heatmaps [60]. All analyses were conducted within the environment of R to ensure reproducibility and accuracy.

## 3. Results

### 3.1. Study Site Characteristics

The 60 soil samples used in this investigation came from three different sites in the northern area of the Tabuk region, at different depths, from 10 m to 20 m. The geographic coordinates of these habitats show that they are geographically dispersed, with median values of pH 7.2 and 8.3, respectively. There were also differences in temperature of 28.5 °C (range: 28.4 °C to 29.8 °C). Further, the turbidity range was from 11 to 16, and significant variations were observed in the salinity range between 43.0 and 44.4. An overview of the features of the study site is given in Table 1.

### 3.2. Taxonomic Composition

**Figure 1:**
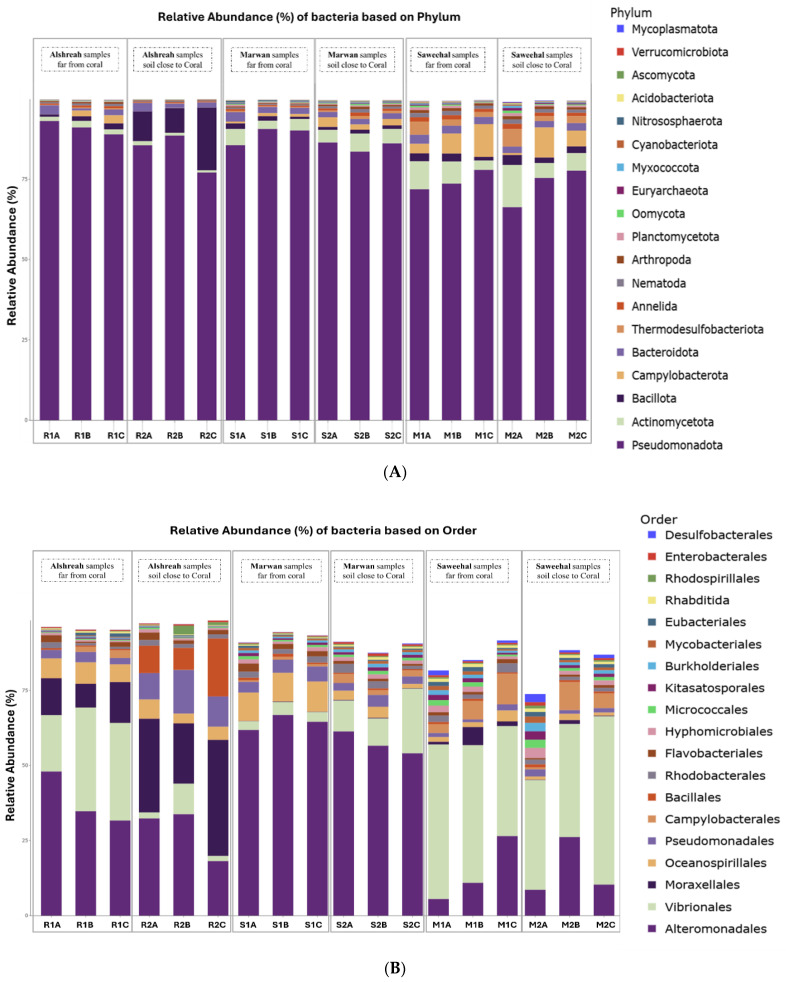

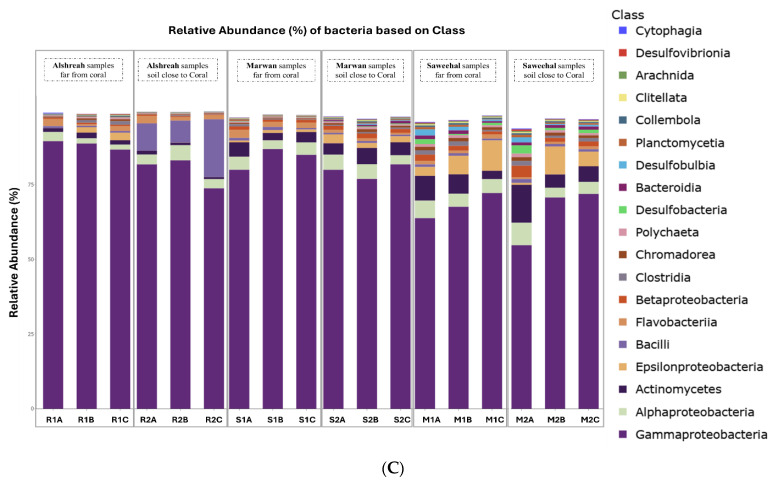
The relative abundance of the main bacterial phyla (**A**), orders (**B**), and classes (**C**) in soil samples taken from three different localities in the northern Red Sea—Alshreah, Saweehal, and Marwan—at different distances from coral reefs. Pseudomonadota, Actinomycetota, and Bacillota were significantly enriched at the phylum level (**A**), whereas Alteromonadales, Vibrionales, and Moraxellales were the most common bacterial orders (**B**). The three most prevalent groupings at class level (**C**) were Actinobacteria, Gammaproteobacteria, and Alphaproteobacteria. These differences demonstrate how environmental variables and coral closeness affect the spread of microorganisms in various settings.

**Figure 2:**
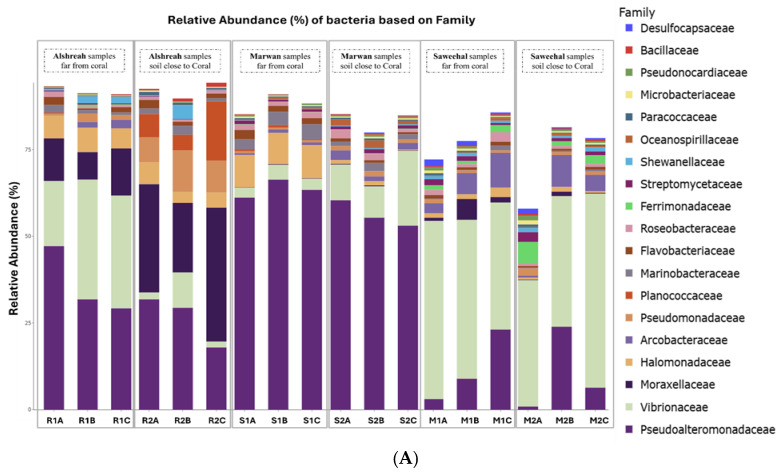

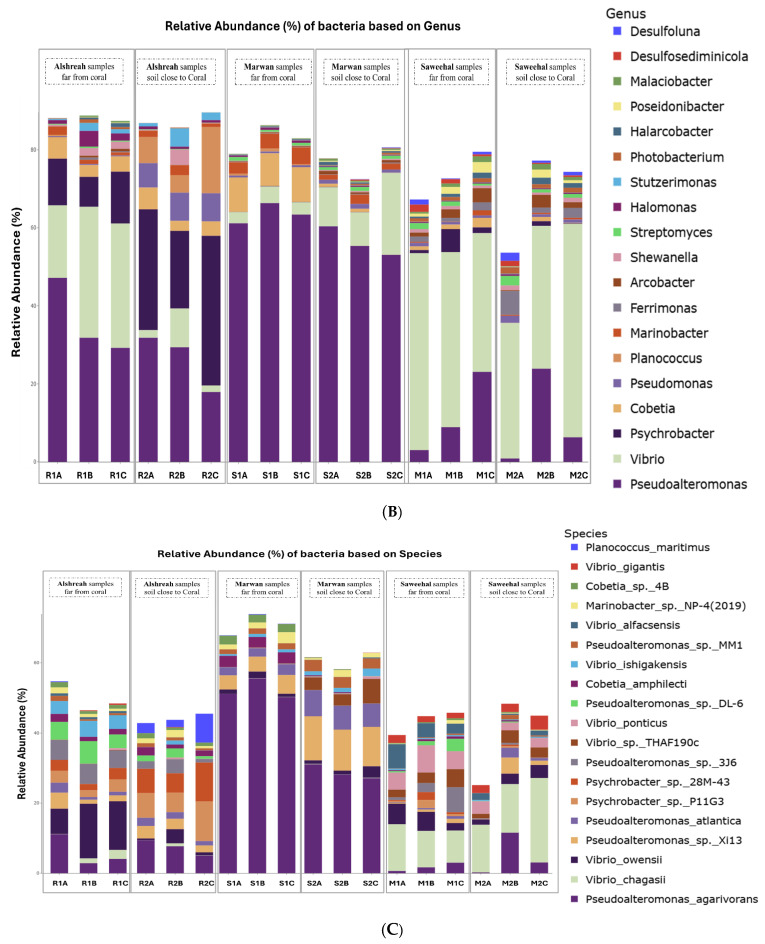
Relative abundance of dominant bacterial family (**A**), genus (**B**), and species (**C**) in soil samples from three different locations, close and far from corals, in the northern Red Sea—Alshreah, Saweehal, and Marwan. The predominant families were Pseudoalteromonadaceae, with *Vibrionaceae* and Moraxellaceae following closely (**A**). At the genus level, the most prevalent were *Pseudomonas*, *Vibrio*, and *Psychrobacter* (**B**). In terms of species, *Pseudoalteromonas agarivorans*, *Vibrio chagasii*, *Vibrio owensii*, and *Pseudoalteromonas* sp. Xi13 were the most abundant (**C**).

Metagenomic sequencing identified a total of 12,299 operational taxonomic units (OTUs), which were classified into 73 phyla, 149 classes, 322 orders, 736 families, and 2545 genera. At the phylum level, Pseudomonadota dominated the microbial communities across all samples, followed by Actinomycetota and Bacillota. The most abundant orders were Alteromonadales, followed by Vibrionales and Moraxellales. At the class level, Gammaproteobacteria, Alphaproteobacteria, and Actinobacteria were the most prevalent. The dominant families included Pseudoalteromonadaceae, followed by *Vibrionaceae* and Moraxellaceae. At the genus level, *Pseudomonas*, *Vibrio*, and *Psychrobacter* exhibited the highest relative abundances. Finally, the most abundant species were *Pseudoalteromonas agarivorans*, *Vibrio chagasii*, *Vibrio owensii*, and *Pseudoalteromonas* sp. Xi13. The relative abundances of dominant bacterial taxa at different taxonomic levels are visually represented by phylum (Figure 1A), order (Figure 1B), class (Figure 1C), family (Figure 2A), genus (Figure 2B), and species (Figure 2C).

### 3.3. Dominant Microbial Genera per Sample

**Alshreah Samples:** Far from Coral (R1A, R1B, R1C): The dominant genus in these samples is *Pseudoalteromonas*, averaging 36% of the microbial population. Other notable genera include *Vibrio* at 28% and *Psychrobacter* at 11% Table 2. These genera reflect a marine-associated microbiome characteristic of areas further from coral influence. Alshreah Samples Close to Coral (R2A, R2B, R2C): *Psychrobacter* dominates these samples, accounting for about 29%, followed by *Pseudoalteromonas* (26%) and *Vibrio* (8%) Table 2. The change in dominance reflects that the microbial communities are responding to proximity to coral, which could affect nutrient cycling.

**Saweehal Samples:** Away from Coral (S1A, S1B, S1C): *Pseudoalteromonas* leads again, averaging 63%. *Vibrio* contributes about 3%, and *Cobetia* 9% Table 2. These results indicate a microbiome very much affected by the open-water environment. Saweehal Samples Close to Coral (S2A, S2B, S2C): The community is slightly diversified but dominated by *Pseudoalteromonas* at 57%, with minor contributions from *Vibrio* (8%) and *Cobetia* (7%) Table 2. Such an indication shows coral influence, possibly contributing to the higher presence of symbiotic or coral-associated microbes. 

**Marwan Samples:** Far from Coral (M1A, M1B, M1C): The dominant genus is *Vibrio* at an average of 44%, followed by *Pseudoalteromonas* at 13% and *Psychrobacter* at 2% Table 2. This shows a different microbial profile, probably affected by other environmental factors. Marwan Samples Close to Coral (M2A, M2B, M2C): *Vibrio* still dominates at 42%, while *Pseudoalteromonas* accounts for 10% and *Psychrobacter* 2% Table 2. The data suggest that, although proximity to coral alters the community structure, *Vibrio* is still a resilient genus under these conditions.

### 3.4. Analysis of Bacterial Communities in Coral Proximity and Distances

**Figure 3:**
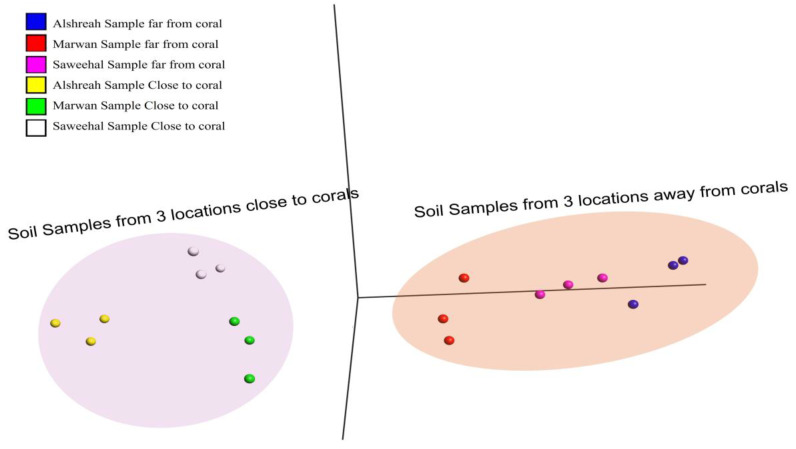
PCA of bacterial community composition. This figure depicts how samples cluster by bacterial community composition using PCA. The samples are color-coded according to their distance from coral (far-from-coral vs. close-to-coral) and grouped according to location (Alshreah, Saweehal, and Marwan).

To understand the relationships of samples based on bacterial community composition, PCA (Principal Component Analysis) and pairwise correlation analyses were performed by using species prevalence data. The obtained results showed significant clustering patterns, as samples closer to corals formed distinct clusters separate from those farther away (Figure 3). These clusters, thereby, indicate how proximity to corals influences the bacterial communities, perhaps due to ecological or environmental factors forcing microbial associations. The PCA in Figure 2 shows that samples closer to coral were more similar to each other (FDR-corrected *p*-value = 7 × 10^−5^).

Further analysis with 2D hierarchical clustering corroborated the PCA results. Samples differentiated using the distance from coral presented separated bacterial genera (Table 2)

Dominant species in samples closer to coral included *Pseudoalteromonas_agarivorans* (30%), *Vibrio_owensii* (11%), and *Pseudoalteromonas*_sp._Xi13 (10%), indicating a coral-associated microbiome. Conversely, samples farther from coral were dominated by *Pseudoalteromonas*_sp._DL-6 (35%), *Vibrio_chagasii* (13%), and *Psychrobacter*_sp._P11G3 (11%).

**Figure 4:**
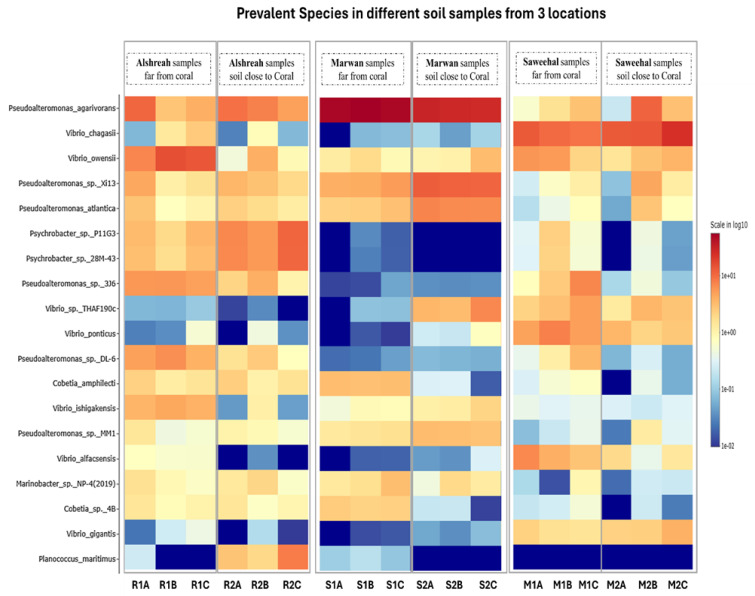
Heatmap of bacterial abundance across samples. Figure 2 shows the top bacterial species’ relative abundance across all samples, through a 2D hierarchical clustering heatmap. In the heatmap, rows represen different bacterial species, whereas the columns correspond to the samples grouped together based on their proximity to the coral and location.

In every group, unique populations of bacteria were present. For instance, in Alshreah samples far from coral, the *Pseudoalteromonas_agarivorans* and *Vibrio_owensii* were dominant, while the near-coral samples showed a higher relative abundance of *Pseudoalteromonas*_sp._Xi13. Likewise, Saweehal samples far from coral had dominance of *Pseudoalteromonas_atlantica* and *Cobetia*_sp._cqz5-12. Such differences point out how the environment in association with coral dictates microbial communities. Pairwise correlation analysis revealed significant associations among bacterial species. Strong positive correlations were observed between genera *Pseudoalteromonas* and *Vibrio* in close-to-coral environments, suggesting that these bacteria may play a synergistic role in nutrient cycling or coral health. In contrast, genera like *Psychrobacter* and *Cobetia* were predominant in nutrient-sparse conditions of open-water environments. The heatmap (Figure 4) visually supports these findings: samples closer to coral are associated with higher microbial diversity, while samples farther away are more homogenous. The PCA plots in Figure 3 also depict the spatial separation of clusters with distinct community structures, indicating different community structures that are dependent on proximity to coral (FDR-corrected *p*-value = 5 × 10^−15^). The relative abundance of the top 10 bacterial species (Table 3) was then analyzed across Alshreah, Saweehal, and Marwan samples, distinguishing between those collected far from the coral and those close to it. There were significant differences in microbial community composition in relation to the proximity to coral.

### 3.5. Microbial Alpha Diversity

To estimate the microbial diversity in each sample, we applied a series of alpha diversity indices (Table 4). Observed species richness is the number of different OTUs that exist, and it displayed large variability among samples, being as low as 3069 OTUs to as high as 12,217 OTUs. The species richness estimators, Chao1 and ACE, supported these findings and revealed that the samples from Alshreah (R1A, R1B, and R1C) and Saweehal (S1A, S1B, and S1C) possessed the highest diversity, especially in Saweehal Far with 52.28% and Alshreah Close with 7.35%.

To account for both species’ richness and evenness, we further calculated the Shannon and Simpson indices. These indices exhibited generally moderate to high diversity among the samples, with the highest marked diversity being recorded at Alshreah Close (7.35%). The Fisher’s alpha index that estimates species richness showed an agreement in the trend with the abovementioned indices and highlighted samples from Saweehal Far and Alshreah Close as the ones most diverse in microbial communities, while Marwan Far showed diversity metrics at lower ranges every time. In contrast, samples from Marwan Far (1.75%) and Alshreah Far (5.99%) had relatively lower values for all indices, indicating a less diverse microbial community. These results indicate significant variation in microbial diversity among the different locations and environments, which suggests that proximity to coral and geographical factors influence microbial community structure. Table 4 presents the alpha diversity metrics that were observed for the analyzed samples.

### 3.6. Clusters of Orthologous Groups (COG) Functional Annotation

To explain the functional profiles of protein clusters in samples from different proximities to coral, COG functional annotation was performed. A hierarchical classification of COG functions (Supplementary Materials) was established based on the average TPM (Transcripts Per Million) values of clustered proteins assigned to each COG category across the far-from-coral and close-to-coral samples.

### 3.7. COG Functional Categories in Far-from-Coral Samples

Of the top 20 COG categories in the far-from-coral samples, the most abundant functional annotations were as follows:

- COG0840: Methyl-accepting chemotaxis protein: This category was present prominently in all the far-from-coral samples, averaging 6000–7147 TPM, which means that this category might be responsible for the adaptation and movement of microorganisms according to environmental stimuli.
- COG0841: Cation/multidrug efflux pump: This functional class showed the highest average TPM values across the far-from-coral samples, ranging from 5358 to 7147. It may reflect involvement in efflux functions and, as such, might be responsible for contributing to resistance in microbial communities of nutrient-limited or stress environments.
- COG0642: Signal transduction histidine kinase: COG group with function related to signal transduction and mechanisms responding to the environment; all have high TPM values; this average is 5051–5533. The presence of such proteins might be indicative of microbes’ adaptation to changing conditions.
- COG1629: Outer membrane receptor proteins, primarily Fe transport: In high abundance in the far-from-coral samples (average TPM = 4200–4533), these proteins can presumably participate in nutrient acquisition, particularly iron, since iron is a limiting element for marine environments.

### 3.8. COG Functional Categories in Close-to-Coral Samples

In Close-to-Coral Samples, the Following Categories of COG Were Dominant:

- COG0841: Cation/multidrug efflux pump: The category of COG0841, like far-from-coral samples, dominated close-to-coral samples, with TPM ranging from 4165 to 7543. These efflux pumps could be contributing toward cellular integrity and environmental responses, possibly in association with defense mechanisms mediated by coral-associated microbes.
- COG0840: Methyl-accepting chemotaxis protein: Proteins in this category were also highly represented (average TPM = 4338–6524), which reflects their participation in microbial responses to chemical gradients and signaling in the coral-associated environment.
- COG0642: Signal transduction histidine kinase: This category showed a high level of abundance (average TPM = 4220–5166) in coral-associated samples, indicating active regulation of the microbial signaling pathways in the presence of the coral ecosystem.
- COG1012: NAD-dependent aldehyde dehydrogenases: These metabolism and detoxification enzymes were significantly enriched in the near-coral samples (average TPM = 4347–4649), reflecting the metabolic shifts microbes might undergo in response to coral-driven nutrient environments.
- COG0243: Anaerobic dehydrogenases, usually selenocysteine-containing: This functional category has TPM values of about 4124, and this would reflect the microbial adaptation to oxygen-limited environments that could be a reflection of anaerobic or microaerophilic conditions near the coral.
- COG0841/COG0840: The most abundant functional categories between the two environments were COG0841 (Cation/multidrug efflux pump) and COG0840 (Methyl-accepting chemotaxis protein), indicating their key role in microbial survival and adaptation to different environmental conditions. Other important functions include signal transduction (COG0642), which plays a role in response to environmental stimuli, and iron transport (COG1629), which is potentially crucial for nutrient acquisition in both environments. These results give a broad view of the functional roles of microbial communities in both far-from-coral and close-to-coral samples, with key biological processes such as stress response, nutrient transport, and environmental signaling.

## 4. Discussion

Coral reefs are complex ecosystems that are essential to preserving the health of the seas. However, a number of recent studies offer strong evidence that interactions between microbes and corals are not always consistent throughout the colony and are instead influenced by a variety of factors, such as the physical environment, life history stage, host physiological characteristics, and location within the coral substructure (tissue, gastric cavity, mucus, and skeleton) [61]. Numerous studies have demonstrated that coral microbial communities are frequently species-specific and that microbial communities within a species can be extremely stable across biogeographies and environmental conditions. Microbes are essential to several reef activities, including primary production and the cycling of nutrients and organic waste [61,62].

Microbes are pervasive symbionts of eukaryotic species, supplying the host with nutrition, facilitating chemical cycle, and offering defense roles. They have an effect on the coral’s surface area and total accretion, but they also have an impact on community dynamics, coral reproduction, reef species diversity and prosperity, and structural topography [61]. Common coral associates known as ciliates are believed to top-down regulate particular microbial populations and opportunistically feed on coral-associated bacteria. Corals are disease resistant because they have inherent immune systems; coral mucus produces antibiotics and utilizes a cellular phagocytic defense mechanism to eliminate the infectious bacteria and defend against pathogens [62].

**Figure 5:**
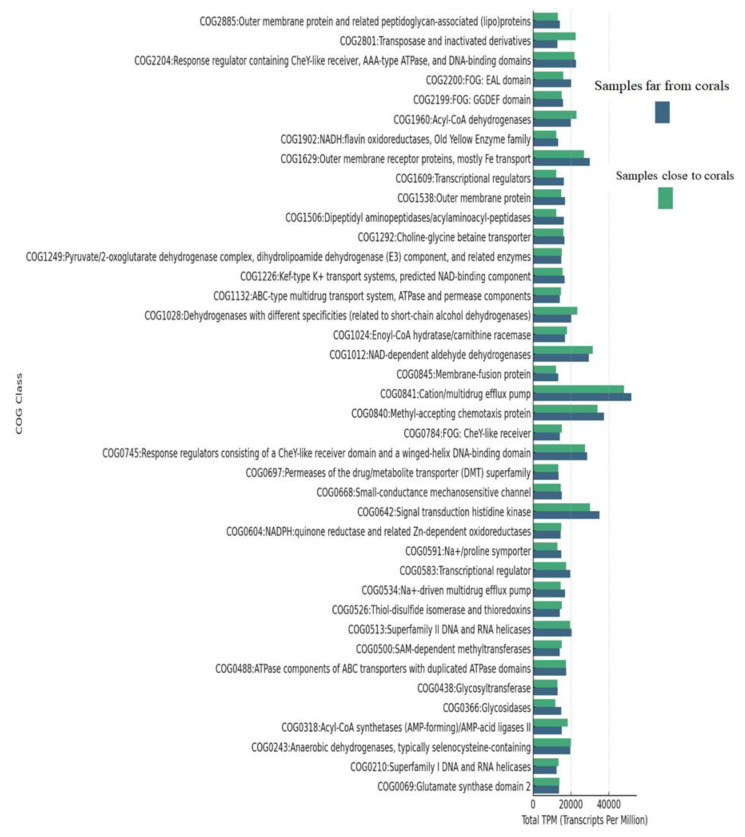
Bacterial Communities in Coral Proximity and Distances.

The Red Sea has some unusual features that may make microbial processes and interactions unique there. These include very high temperatures, high salinity (from very little freshwater input), very high solar irradiation all year round, and a large amount of dust from the nearby deserts that comes in through the Aeolian process. Despite the Red Sea’s exceptional biotic conditions, research into the sea has hitherto been hindered by political and practical hurdles. The majority of early studies were carried out in the Gulf of Aqaba, a tiny, shallow outflow in the Red Sea’s northeast (Figure 5).

Research in this domain has yielded significant insights on Red Sea microorganisms, particularly concerning possible disease-associated bacteria of reef creatures, *Corallasia halofolliculina* [45,46,47,48]. In the Gulf of Aqaba, the microorganisms that live in the sand were also seen to change when the sediment depth changed. Although the richness of bacterial communities is similar in surface (0–2 cm), intermediate (2–6 cm), and deeper (6–12 cm) sands, there are taxonomic alterations associated with sediment depth [46]. *Rhodobacteraceae* sequences were found in the more aerobic upper layers of sediment, whereas *acidobacteriales* predominated in the lower and intermediate layers of sediment, perhaps because of the low oxygen levels [47]. From investigations of additional reef sand environments, bacteria from these same families were found [48]. So far, Red Sea sediment investigations have not incorporated archaea in their techniques, but sequences associated with the archaea, and notably the ammonia oxidizer *Nitrosopumilus maritimus*, seem to be common in reef sands [63]. Diseases in coral reefs are caused by a number of factors, including red tide outbreaks, increased seawater depth, excessive solar radiation, rising seawater temperatures, and the presence of human waste [64].

The results of our metagenomic study on coral reef provide baseline information on the prevalence of coral diseases that are having an impact on reefs in the northern region of the Red Sea of Tabuk, Saudi Arabia. This study’s findings unequivocally demonstrate that illness frequency varied greatly among reefs. Disease development may be influenced by both biotic and abiotic factors. Biologic diseases are caused by pathogenic microorganisms, which include bacteria, fungi, and viruses. Abiotic illnesses can arise as a result of environmental stresses that are either naturally occurring or man-made, such as alterations in the surrounding environment or exposure to pollutants.

The metagenomic analysis of the bacterial community structures among different locations of Northern Red Sea Tabuk, K.S.A, identified a total of 12,299 operational taxonomic units (OTUs), which were classified into 73 phyla, 149 classes, 322 orders, 736 families, and 2545 genera. Our study demonstrated the prevalence of the three top phyla: Pseudomonadota, followed by *Actinomycetota* and *Bacillota* across all samples collected from three different sites, Alshreah, Saweehal and Marwan, of the Northern Red Sea coral reef of Tabuk. The five most prevalent bacterial genera among six sample classes collected from three distinct sites (Alshreah, Saweehal, and Marwan) are shown in Table 1, along with their average relative abundance (as a percentage). Samples were collected in three repetitions from two habitats at each location, obtained far from coral and samples taken near to coral. Samples of Alshreah that are far from coral (R1A, R1B, and R1C): with an average of 36% of the microbial population, *Pseudoalteromonas* is the most prevalent genus in these samples. Other noteworthy genera are *Psychrobacter* (11%) and *Vibrio* (28%). The marine-associated microbiome found in regions farther away from coral impact is reflected in these taxa (Table 1). These results are quite similar to previous studies conducted in the Bohai, Yellow, South, and East China Seas, which have shown that the high abundance of Pseudomonadota and *Plancytomycetes* could be due to environmental eutrophication [65,66].

Soil samples of Alshreah Near Coral (R2A, R2B, R2C) had almost 29% of the *Psychrobacter predominates*, followed by Pseudoalteromonas (26%) and *Vibrio* sp. (8%). The shift in dominance indicates how the microbial communities react to coral proximity, which may have an impact on the cycle of nutrients. *Planctomycetota* have significant biogeochemical functions in anaerobic ammonium oxidation [67], methane oxidation [68], and carbon recycling [69], whereas *pseudomonadota* are essential for the breakdown of organic nitrogen in sediments [70,71]. Since both phyla are chemotrophic bacteria, their high abundance may be a result of the environment’s high relative chemical concentration. For Saweehal soil samples away from coral, with an average of 63%, *pseudoalteromonas* is once again in the lead. About 3% comes from *Vibrio* sp. and 9% from Cobetia. These findings suggest that the open-water environment has a significant impact on the microbiota. Soil samples from the same site from the vicinity of the coral reef account for 57% of the *Pseudoalteromonas* total, with *Vibrio* and *Cobetia* contributing just 8% and 7%, respectively (Table 1). These results demonstrate the effect of corals, which may be one reason why symbiotic or coral-associated bacteria are more prevalent. According to current research, there are bacterial species present that are assumed to play advantageous functions for the deep Red Sea corals, such as those related to the nitrogen and carbon cycles, e.g., E. fistula, *Dendrophyllia* sp [72]. According to earlier studies, members of the Pseudomonadaceae and *Endozoicomonadaceae* families have a high abundance of genes encoding proteins involved in host–symbiont identification and colonization [61].

Previous studies also suggest other characteristics, such as the breakdown of host-derived taurine, which was commonly observed in strains of *Cobetia* and *Halomonas* (Halomonadaceae), and antiviral protection in some *Endozoicomonas* strains. The widespread occurrence of these bacterial species linked to various coral taxa raises the possibility that they are significantly corroborated for coral probiotic thermal protective roles, metabolism, and health [73,74]. Some studies provided evidence that some members of this taxon play a key role in the sulfur cycle in corals [75]. Metagenomic profiling revealed that some other samples had a variety of metabolic sources, while around half of the samples indicating possible concentrations of high sulfate and nitrite reducers and dehalogenating *bacteria*. The dominance of *Alteromonas* and *Pseudoalteromonas* is known to be important in the metabolism of dimethyl-sulfoniopropionate (DMSP), which may be the only explanation for the high concentration of sulfate reducers [76]. Elevated levels of DMSP production in Northern Red Sea corals and nearby waters may be indicated by the high abundance of certain taxa that can prove to be good beneficial microorganisms for corals (BMC) candidates, and we found the genes involved in the degradation of dimethyl sulfoniopropionate (DMSP) in the genomes of *Endozoicomonas*, verifying that some bacteria can use DMSP as a carbon source [77]. *Psychrobacter Phenylpyruvicus* is a nonmotile, Gram-negative, catalase- and oxidase-positive bacterium that is easily isolated from saltwater and other typical marine settings. It may be involved in the degradation of dimethylsulfoniopropionate (DMSP), a significant osmolyte of algae. When DMSP is broken down, dimethylsulfide is released into the atmosphere, where it may affect the weather by promoting the production of clouds. Although *P. pacifiensis* may also be found in subseafloor sediments and at sea bottoms of up to 6000 m, most soil habitats cannot support *Psychobacter* species unless they are exposed to constant low temperatures and sporadic freezing [77].

The bile acid derivatives from marine *Psychrobacter* sp. were described as an example of marine-derived antibacterial steroids in previously conducted research [78]. Both the coral and the endosymbiotic dinoflagellates create DMSP, which is then broken down by related bacteria to yield acrylate, dimethylsulfide (DMS), and dimethyl sulfoxide (DMSO). These compounds may play a part in antioxidant capacity and osmoregulation [79]. Therefore, increased DMSP production might indicate the ability to endure the extreme salinity and heat anomalies within the distinctive nature of the Northern Red Sea. In our studies, different species of Vibrio bacteria such as *Vibrio owensii*, *Vibrio chagasii*, and *Vibrio ponticus* are shown in Table 2. Species of vibrio owansii OCN002 have been recognized as the first bacteria from Hawaii’s reefs that cause chronic montipora white syndrome (cMWS), a tissue-loss disease found on corals throughout the Hawaiian archipelago. Another bacterial species of *Pseudoalteromonas piratica* strain OCN003 causes chronic MWS to acute MWS (aMWS) [80]. Pseudoalteromonas agarivorans species produce the most common metalloproteinase collagenolytic protease, which degrade collagen and is the primary pathogen affecting the coral reef [68]. Some species of *Pseudoaltromonas* exhibit extracellular antibacterial compounds produced by *Pseudoalteromonas* spp., linked to certain coral species that may protect against invading pathogens. Similarly, other studies have reported a higher prevalence of *Pseudoalteromonas* phylotypes in *Acropora millepora* that are resistant to *Vibrio* sp. infection. Additionally, certain strains of *P. alteromonas* species are known to fix nitrogen through dinitrogen, and they may then transfer fixed nitrogen to the algal endosymbiosis linked to *P. damicornis larvae* [81]. Furthermore, the Red Sea possesses a high level of coral endemism, but research on endemic species is still in its early stages. There is a chance to investigate endemic corals that could have unique characteristics, as we were unable to locate any relevant publications about the bacterial communities of Red Sea-endemic corals. These particular characteristics and possible special bacterial partners may be especially intriguing if linked to the endemic corals’ ability to adjust to environmental gradients in the Red Sea through the flexibility and plasticity of their microbiomes [82]. Additionally, it has been reported that *Vibrios* microbes can produce enzymes to break down polycyclic aromatic hydrocarbons to produce antibiotic substances and provide essential polyunsaturated fatty acids. Chitin is the second most abundant polymer and the primary source of amino sugars in the oceans [83]. Salini *vibrio* sp., a member of the *Vibrionaceae* family, was part of a probiotic consortium that helped coral bleaching recover and protect against mortality, according to Santoro and colleagues [84]. Because of its antagonistic activity against two well-known coral diseases, *Vibrio coralliilyticus* and *V. alginolyticus*, this *Salinivibrio* sp. was chosen to be a part of the consortium of beneficial microorganisms for corals (BMCs). The distinct functions of particular genera within the same bacterial family in the coral holobiont can be explained by the substantial differences in genomes between non-pathogenic and pathogenic members of the *Vibrionaceae* family [61]. The studies indicate that while the precise function of various bacterial phylotypes in Red Sea corals is yet to be fully understood, they may play crucial roles in holobiont fitness. This study found distinct bacterial communities in each group, with *Pseudoalteromonas agarivorans* and *Vibrio owensii* dominating in Alshreah samples distant from coral, while *Pseudoalteromonas*_sp._Xi13 was more abundant in closer samples. Most of the microorganisms found in coral mucus are *Vibrio* strains, and the quantities of these bacteria are significantly greater than those found in the surrounding saltwater. The majority of *Vibrio* strains, including *V. mediterranei*, *V. coralliilyticus*, *V. harveyi*, and *V. alginolyticus*, are the cause of coral bleaching and have been shown to induce illnesses in marine organisms when their mucus separates from the coral [85]. In coral reef environments, some *Vibrio* species are essential for the transfer and circulation of a variety of materials, such as chitin, alginate, and agar and energy, as these Vibrio genera have a tendency to consume an extensive array of carbon substrates. It was shown that coral symbiotic microorganisms of Proteobacteria, such as *Pseudoalteromonas*, *Pseudomonas* and *Cyanobacteria*, were the most prevalent phyla of coral microorganisms and are crucial markers of coral health in maintaining material transformation and biogeochemical cycles.

In habitats around coral, strong positive relationships between the species *Pseudoalteromonas* and *Vibrio* were found, indicating that these bacteria could work in concert to support coral health or nutrient cycling. Therefore, corals and the nearby saltwater could be the best places to research *Vibrio*’s spread and pathogenicity as well as find new species and enzymes. Furthermore, Figure 4 depicts the bacterial abundance across all samples, through a 2D hierarchical clustering heatmap. The heatmap’s rows represent the various bacterial species, while the columns show the samples that have been grouped together according to their site and closeness to the coral. The Shannon and Simpson indices were also computed to take species richness and evenness into consideration. The highest diversity was identified in Saweehal far from corals (S1A, S1B, and S1C) (52.8%) and in Alshreah close (7.35%), while Marwan Far showed lower ranges of microbial community diversity every time, i.e., 1.75%. The microbial community was less diversified in the samples from Alshreah Far (5.99%) and Marwan Far (1.75%), which had comparatively lower values for all indices. According to these findings, there is a notable difference in microbial diversity across the various settings and locales, revealing that geographic variables and coral closeness affect the diversity of microbial communities (Table 3). The more prevalent microbial community of corals from inner and outer reef zones found in the soil of various coral reef settings is consistent with the *alphaproteobacterium Candidatus* having a higher relative abundance in the surface mucus layer (SML) microbiome of corals around the coral reef zone.

The taxonomic diversity measures support the idea that the microbial communities in the coral’s immediate closest environment influence the coral SML microbiome. However, both at the taxonomic and functional levels, the local environment (i.e., inner reefs vs. outer reefs) and the coral host versus soil simultaneously determine the microbial community structure (i.e., relative abundances of sequences) in the Northern Red Sea reef system [73]. Aligning query gene or protein sequences with COG database sequences, COG functional annotation was used to describe the functional patterns of protein clusters in samples that were at varying distances from coral. The average TPM (Transcripts Per Million) values of clustered proteins allocated to each COG category across the far-from-coral and close-to-coral samples were used to create a hierarchical categorization of COG functions (Supplementary Materials). With an average of 6000–7147 TPM, Methyl-accepting chemotaxis protein was found in all of the distant coral samples. This suggests that this category may be the reason for microorganisms’ ability to adapt and migrate in response to environmental stimuli. Methyl-accepting chemotaxis proteins in coral-associated environments were highly represented, depicting their involvement in microbial responses to chemical gradients and signaling.

The multidrug/cation efflux pump, with high average TPM levels, may contribute to microbial resistance in stressful or nutrient-limited settings, as indicated by far-from-coral samples. Similar to the far-from-coralcation, the multidrug efflux pump category dominates close-to-coral samples, supporting environmental reactions and cellular integrity through defensive mechanisms mediated by coral-associated bacteria. Signal transduction histidine kinase has high TPM levels of 5051–5533, suggesting microorganisms have adapted to their far coral environment. Signal transduction histidine kinase abundance in coral-associated samples indicates that the coral ecosystem actively regulates microbial signal transduction pathways and adapts a broad range of environmental cues. Histidine kinase (HK) autophosphorylating response regulator protein enables the cells to detect and react to external stimuli, which thrive as a molecular switch that regulates a variety of effector activities with adaptable conserved domains that may be easily changed to the unique requirements of various systems [86]. Outer membrane receptor proteins, abundant in far-from-coral samples with a TPM average 4200–4533, may be involved in nutrient acquisition, particularly iron, as iron is a limiting element for marine habitats [87].

Soils around the coral reefs are rich in metabolism, with detoxifying enzymes like NAD-dependent aldehyde dehydrogenases undergoing metabolic changes based on coral-driven food conditions. Anaerobic dehydrogenases, including selenocysteine, exhibit microbial adaptability to oxygen-limited conditions, possibly due to anaerobic or microaerophilic conditions near coral, as indicated by TPM values of around 4124. To summarize, the most prevalent COG in both distant and close coral reef environments rely on cation/multidrug efflux pump, methyl-accepting chemotaxis protein, iron transport, and signal transduction for microbial survival, environmental adaptability, and nutrition absorption [87]. The shedding of carbon-rich mucus by corals may result in chemical hotspots, while the diffusion of methyl-accepting compounds away from the coral surface into the surrounding saltwater creates microscale chemical gradients that extend into the nearby ocean [88]. The findings of the methyl chemotaxis pattern suggest that bacteria connected to the surface of the coral had noticeably more chemotactic capabilities then bacteria away from the coral. Chemotaxis may serve as a behavioral filter that determines the makeup of microbial communities inside particular coral microniches [87,88].

It is unclear what environmental conditions lead to the development of planktonic blooms and how microbial communities interact with one another. To determine the rhythmic pattern (diurnal, monthly, or seasonal) of microbial composition and track changes in these patterns in response to human activities, extensive time-series investigations of Red Sea microbiota across multiple niches are required. Coastal areas of the Red Sea have seen a sharp increase in pollution in recent decades as a result of growing industries and urbanization. Industrial and anthropogenic pollutants discharged directly into the marine environment have the potential to alter the makeup of microbial communities. Phylogenetic analysis of cultured microbial communities isolated from sediments in industrial zones along the Egyptian Red Sea coast was carried out by Mustafa et al. [88]. They investigated distinct microbial communities, mainly oil/hydrocarbon degrading bacteria and a number of human pathogens, including known *Vibrio* and Clostridium species [89].

## 5. Conclusions

The present study emphasized the microbiome taxa and gene forming of the microbiome in three distinct locations surrounding and beneath the coral communities. The structural makeup of the microbial communities varied significantly according to the closeness to the coral. This study revealed that *Pseudoalteromonas agarivorans*, *Vibrio owensii*, and *Pseudoalteromonas* sp. *Xi13* were the most predominant species in samples closer to coral. Moreover, *Vibrio* species were the most prevalent microorganisms in the coral mucus. Furthermore, in close-to-coral habitats, there were strong positive associations between the genera *Vibrio* and *Pseudoalteromonas*, indicating that these bacteria may work in concert to support coral health or nutrient cycling.

## Figures and Tables

**Table 1:** Showing different water parameters recorded in the selected sites prior to sampling of the soil samples.

Water Parameters	Average in Site 1	Average in Site 2	Average in Site 3	Average in Site 4	Average in Site 4	Average in Site 5	Average in Site 6	Average in Site 7	Average in Site 8	Average in Site 9
pH	8.1	8.3	8.0	7.3	8.3	8.4	7.2	8.1	8.4	8.3
Temperature °C	28.7	28.4	29.7	29.3	28.0	29.8	29.4	29.5	28.8	29.6
Turbidity (NTU)	13	14	12	13	13	11	14	15	14	16
DO (mg/L)	3.36	3.54	3.31	3.44	3.72	3.46	3.33	3.34	3.52	3.50
Salinity ppt	44.5	43.3	43.6	44.4	43.0	44.0	44.3	44.2	43.47	44.2

**Table 2:** Distribution of top 5 genera across six sample classes. The table provides the average relative abundance (as a percentage) of the five most dominant bacterial genera across six sample classes obtained from three different locations (Alshreah, Saweehal, and Marwan). At each location, samples are taken from two environments: far from coral and close to coral in three replicates.

Genus	Alshreah		Saweehal		Marwan	
	R1A-R1C (%) Far from Corals	R2A-R2C (%) Close to Corals	S1A-S1C (%) Far from Corals	S2A-S2C (%) Close to Corals	M1A-M1C (%) Far from Corals	M2A-M2C (%) Close to Corals
*Pseudoalteromonas*	36	26	63	57	13	10
*Vibrio*	28	8	3	8	44	42
*Psychrobacter*	11	29	~1	~1	2	2
*Cobetia*	4	4	9	7	3	2
*Pseudomonas*	~1	7	~1	~1	3	4

**Table 3:** This table presents the relative abundance (%) of the top 10 bacterial species across samples collected from three different locations (Alshreah, Saweehal, and Marwan) under two environmental conditions: far from coral and close to coral. Columns for “Far-from-Coral (%)” and “Close-to-Coral (%)” provide aggregated averages for the respective environmental conditions across all locations. The table highlights variations in species composition influenced by coral proximity.

Species	Alshreah Far (%)	Alshreah Close (%)	Saweehal Far (%)	Saweehal Close (%)	Marwan Far (%)	Marwan Close (%)	Far-From-Coral (%)	Close-to-Coral (%)
*Pseudoalteromonas agarivorans*	5.99	7.35	52.28	28.77	1.75	4.94	20.00	13.6
*Vibrio chagasii*	1.35	0.31	0.05	0.1	10.98	17.2	4.12	5.87
*Vibrio owensii*	12.24	1.8	1.34	1.79	4.49	2.73	6.02	2.10
*Pseudoalteromonas* sp. *Xi13*	2.47	2.84	4.55	11.84	0.71	1.97	2.57	5.55
*Pseudoalteromonas atlantica*	1.55	1.82	2.58	6.93	0.45	1.2	1.52	3.31
*Psychrobacter* sp. *P11G3*	2.96	7.98	0.02	0	1.07	0.17	1.35	2.71
*Psychrobacter* sp. *28M-43*	2.72	7.86	0.02	0	1.03	0.16	1.25	2.67
*Pseudoalteromonas* sp. *3J6*	5.46	2.4	0.03	0.04	3.5	0.24	2.99	0.89
*Vibrio* sp. *THAF190c*	0.08	0.02	0.06	4.63	3.45	2.59	1.19	2.41
*Vibrio ponticus*	0.21	0.17	0.01	0.39	5.93	2.81	2.05	1.12

**Table 4:** Alpha diversity indices for each sample set.

Sample ID	Observed	Chao1	ACE	Shannon	Simpson	Fisher
Alshreah Far (R1A, R1B, R1C)	5.99	7.35	52.28	28.77	1.75	4.94
Alshreah Close (R2A, R2B, R2C)	5.46	2.4	2.57	5.55	2.99	0.89
Saweehal Far (S1A, S1B, S1C)	52.28	1.75	0.31	2.12	5.87	0.89
Saweehal Close (S2A, S2B, S2C)	4.94	3.31	1.52	5.55	2.77	0.88
Marwan Far (M1A, M1B, M1C)	1.75	4.12	1.75	4.94	2.94	2
Marwan Close (M2A, M2B, M2C)	4.94	1.75	4.94	2.1	0.95	1.1

## Data Availability

Data is contained within the article.

## Supplementary Materials

The following supporting information can be downloaded at: https://www.mdpi.com/article/10.3390/life15030423/s1, Figure S1: (A) A map showing location of the sampling sites within Sweahle. (B) Locations of the sampling sites within Marwan, (C) Location of the sampling site in Alshearh 1 and (D) Location of the sampling site in Alshearh 2. (E,F) Represent the sampling method close to Coral communities and (G) Sampling methods far away from Coral.
