# Supplementary material for: Metagenomic Characterization of Microbiome Taxa Associated with Coral Reef Communities in North Area of Tabuk Region, Saudia Arabia
Source: Life (Basel). 2025 Mar 7;15(3):423. doi: 10.3390/life15030423 (PMC11944186; doi:10.3390/life15030423)
Supplement: Supplementary file 1 [file life-15-00423-s001.zip › life-3426575-supplementary.pdf]

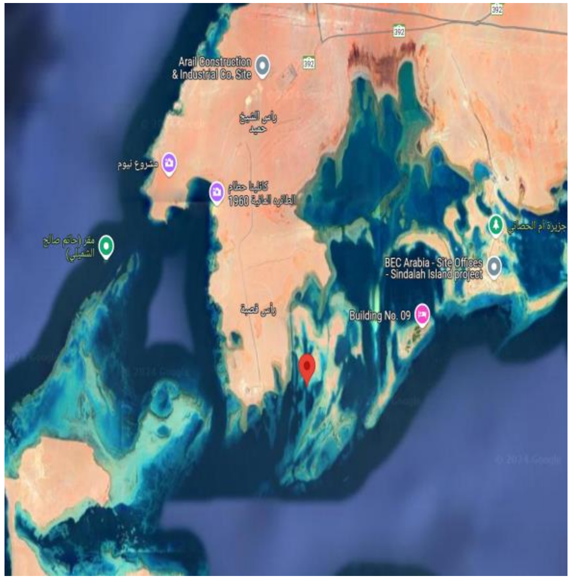
Arail Construction
& Industrial Co. Site
BEC Arabia - Site Offices
- Sindalah Island project
Building No. 09
© 2024 Google

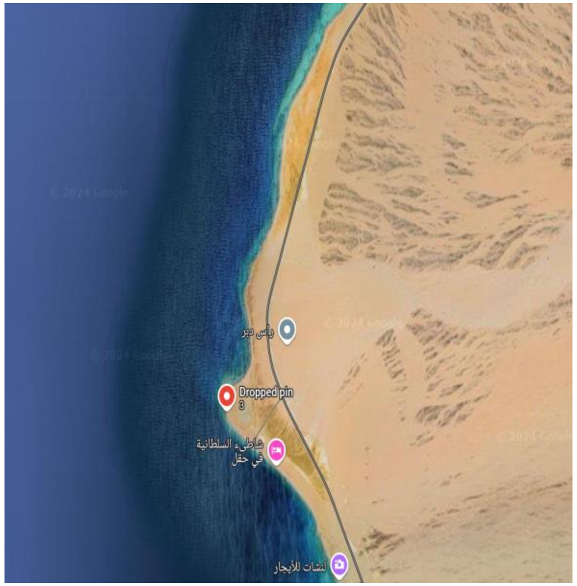
Dropped pin
3
© 2024 Google

(**A**) (**B**)

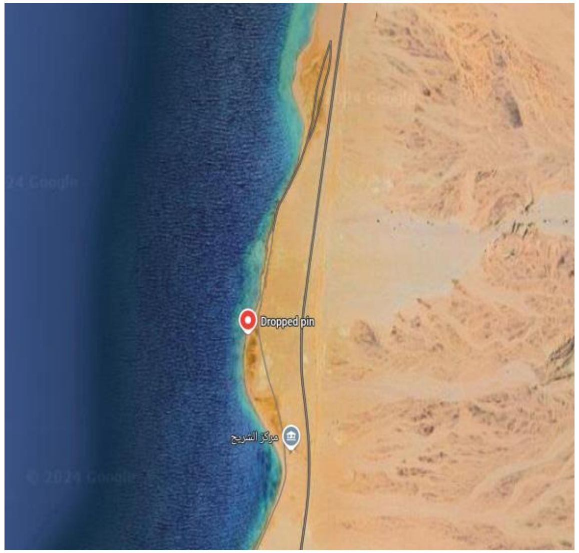
Dropped pin
© 2024 Google

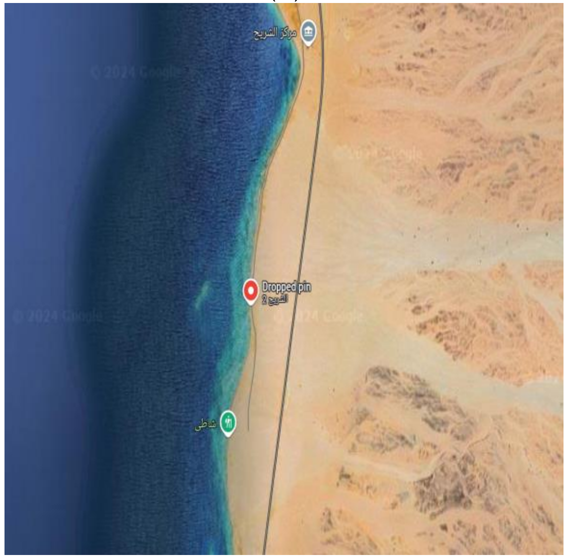
Dropped pin
© 2024 Google

(**C**) (**D**)

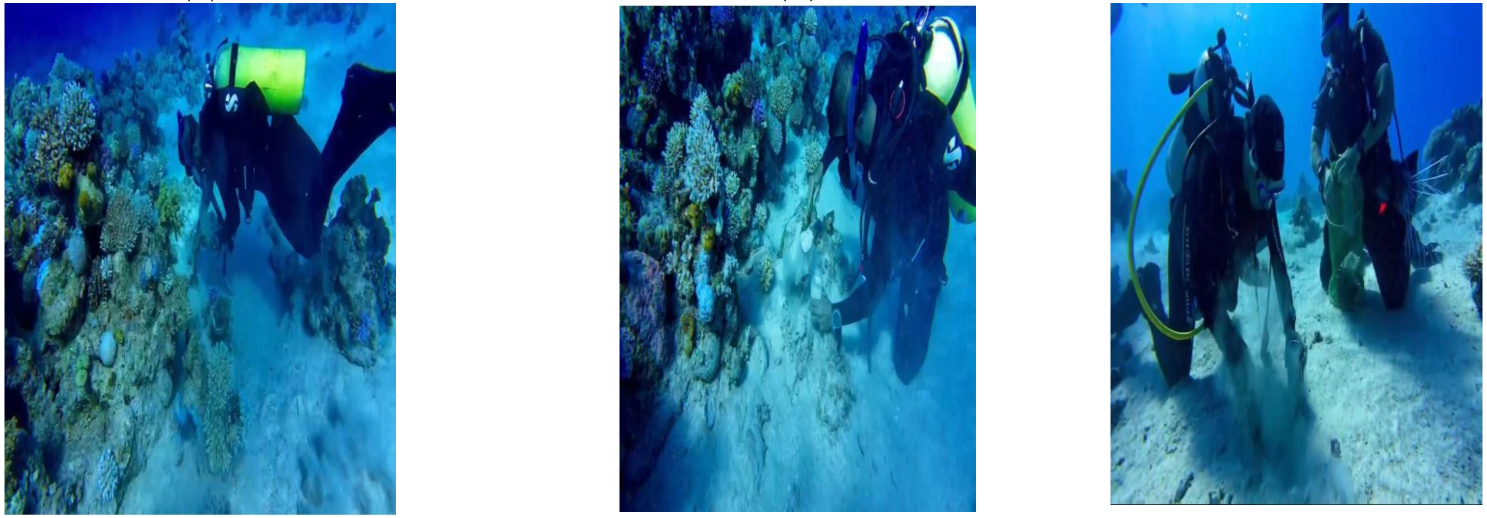

(**E**) (**F**) G

Figure S1. (**A**) A map showing location of the sampling sites within Sweahle. (**B**) Locations of the sampling sites within Marwan, (**C**) Location of the sampling site in Alshearh 1 and (**D**) Location of the sampling site in Alshearh 2. (**E**,**F**) Represent the sampling method close to Coral communities and (**G**) Sampling methods far away from Coral.
